# Retraction: MicroRNA-411 Inhibits Cervical Cancer Progression by Directly Targeting STAT3

**DOI:** 10.32604/or.2024.055030

**Published:** 2024-07-17

**Authors:** Oncology Research Editorial Office

The published article titled “MicroRNA-411 Inhibits Cervical Cancer Progression by Directly Targeting STAT3” has been retracted from *Oncology Research*, Vol. 27, No. 3, 2019, pp. 349–358.

DOI: 10.3727/096504018x15247361080118

URL: https://www.techscience.com/or/v27n3/48598

Following the publication, concerns have been raised about a number of figures in this article. The transwell invasion assays shown in Fig. 5C were strikingly similar to data appearing in different form in other articles written by different authors at different research institutes that had already been published.

The authors were contacted and invited to comment on the concerns raised and to provide the original, unmodified figures, but did not respond. The Editors-in-Chief therefore no longer have confidence in the integrity of the data in this article and decided to retract this article.

All authors have not responded to correspondence about this retraction. As a responsible publisher, we hold the reliability and integrity of our published content in high regard. We deeply regret any inconvenience caused by this situation to our readers and all concerned parties.

